# Reclamation in southern China: The early Chu’s agriculture revealed by macro-plant remains from the Wanfunao site (ca. 1000–770 BCE)

**DOI:** 10.3389/fpls.2022.942366

**Published:** 2022-08-02

**Authors:** Ruichen Yang, Liya Tang, Dong Zhao, Wenxin Huang, Yunbing Luo

**Affiliations:** ^1^China-Central Asia “The Belt and Road” Joint Laboratory on Human and Environment Research, Key Laboratory of Cultural Heritage Research and Conservation, School of Culture Heritage, Northwest University, Xi’an, China; ^2^Yichang Museum, Yichang, China; ^3^Hubei Provincial Institute of Cultural Relics and Archaeology, Wuhan, China

**Keywords:** southern China, Bronze Age, Zhou Dynasty, early Chu culture, compatible environment, multi-crop farming system

## Abstract

The Wanfunao site was a large Chu settlement in Zhou Dynasty. It was located on an alluvial plain along the Yangtze River in the Yichang section. The region around the site comprised mountains, hills, and plains, which was a compatible environment for the cultivation of various crops. Previous studies have suggested that the middle and lower reaches of the Yangtze River are one of the most productive regions for rice cultivation. Besides rice, however, seven dryland crops have been found at the Wanfunao site: foxtail millet, broomcorn millet, wheat, barley, oat, buckwheat, and adzuki bean. Among them, foxtail millet and rice are most ubiquitous. The crop assemblage has revealed that the northern dryland crops, including those were newly adapted cereals such as foxtail millet, wheat, and barley, gradually dispersed southward and became a part of the diet along with rice. This can be attributed to southern Chinese inhabitants’ reclamation of the hilly environment for agriculture. Although communities in southern China had cultivated rice on the plains for thousands of years, newly introduced dryland crops from north China adapted to mountainous environments better. The development of multi-cropping systems in southern China likely involved changes in agricultural ontology associated with the adaptation of northern crops in southern environments newly encountered. Additionally, the assemblage of foxtail millet grain/rice spikelet base in the site may have been used for livestock feeding. A wide range of landforms, compatible farming, and surplus agricultural products for husbandry may have been a part of the economic foundation that facilitated the rise of Chu.

## Introduction

**FIGURE 1:**
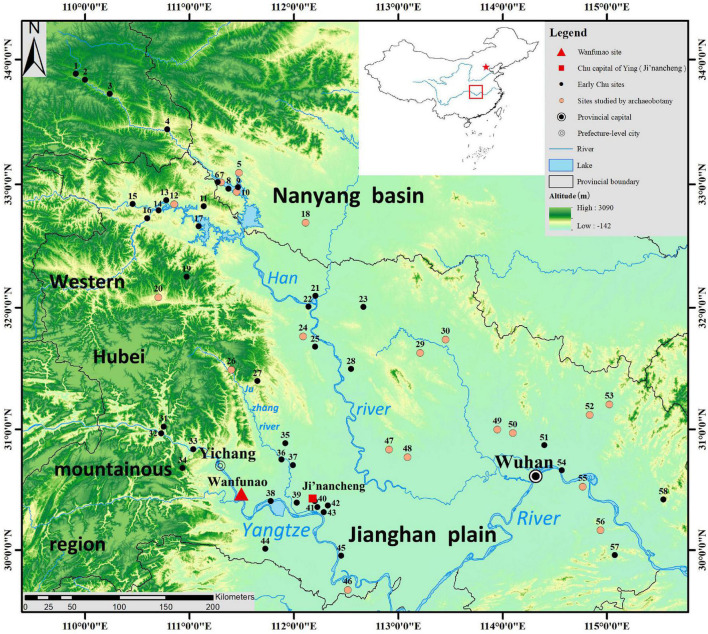
Geographic distribution of the sites of early Chu culture and other related sites studied by archeobotany. (1) Chenyuan, (2) Zijing, Donglongshan, (3) Gongjiawan, (4) Guofenglou, (5) Gouwan, (6) Xiazhai, (7) Shenmingpudong, (8) Shangang, (9) Shuanghezhen, (10) Xiawanggang, (11) Xiajiao, (12) Qinglongquan, Dasi, (13) Qujiawan, (14) Liaowadianzi, (15) Zhongtaizi, (16) Dadongwan, (17) Zhujiatai, (18) Baligang, (19) Sunjiaping, (20) Jijiawan, (21) Xiaojialing, (22) Zhenwushan, (23) Maogoudong, (24) Huanggang, Xiajiangjiabianzi, (25) Guojiagang, (26) Mulintou, (27) Lujiahe, (28) Liuhe, (29) Jizibao, Zhoujiabang, (30) Miaotaizi, Zhoujiazhai, (31) Guanzhuangping, (32) Miaoping, (33) Huangtubao, Tanjiatuo, Xiaoxikou, Shangmonao, (34) Jijiahu, (35) Yangmugang, (36) Fengshan, (37) Fujiayao, Mopanshan, Zhaojiahu, (38) Hejiawa, (39) Meihuaiqiao, (40) Motianling, (41) Zhangjiashan, (42) Yangcha, (43) Zhouliangyuqiao, (44) Boyushan, (45) Jingnansi, (46) Zoumaling, (47) Qujialing, (48) Sanfangwan, Tanjialing, (49) Zhengjiadamiao, Yejiamiao, (50) Chenghuangdun, (51) Lutaishan, (52) Jinluojia, (53) Lishangang, (54) Xianglushan, (55) Chengzishan, (56) Xiezidi, (57) Heshangnao, and (58) Maojiazui.

Chu, a name of great significance in Chinese history, refers to not only a state but also a region. It was one of the Five Hegemonies and Seven Powers during the Eastern Zhou Dynasty and played a significant role in the long history of China. Today, Hubei Province residents in the middle reaches of the Yangtze River occupy the core area of Chu and still consider themselves Chu’s descendants (CENTRAL Hubei–School History Compilation Group of Jingzhou Revolutionary Cadre School, 1993). The Jianghan Plain is located around this area. During the Neolithic period, the Pengtoushan culture (7000–6000 BC), the Chengbeixi culture (6500–5000 BC), the Tangjiagang culture (4800–4300 BC), the Daxi culture (4500–3300 BC), the Youziling culture (4000–3100 BC), the Qujialing culture (3400–2500 BC), the Shijiahe culture (2500–2000 BC), and the Post-Shijiahe culture (2200–1800 BC) existed in chronological order. These cultures were followed by the Shang culture, the Zhou culture, and the Chu culture in the Bronze Age (Han, 2016). The Chu culture has come to be widely studied with archaeological works gradually being carried out on Mopanshan site (Lu, 1984), Jijiahu site (Yang, 1980), Zhaojiahu site (Hubei Yichang Museum and the Department of Archaeology of Peking University, 1992), and others (Figure 1).

The middle reaches of the Yangtze River are a transitional zone between northern and southern China. As early as the initial Western Zhou period, Yi Xiong, Chu’s ancestor who resided in northern China acquired the land around the middle Yangtze River under the enfeoffment system by the King Cheng of Zhou (Sima, 1959). For a long time, Yi Xiong and his successors cohabited the region with the aborigines, and a unique Chu culture gradually evolved (Liu and Yin, 2010). The prosperity of the middle Yangtze River began from the late Neolithic period when the indigenous Qujialing and Shijiahe people flourished (Zhang and Yin, 2011). During that time, extensive interactions had occurred between the middle reaches of the Yangtze River and the middle reaches of the Yellow River (Han, 2020). For example, the typical oblique-belly cup of the Qujialing and the Shijiahe cultures appeared in the Central Plains, Loess Plateau, and other regions (Han, 2002). The emergence of the Post-Shijiahe culture was involved in the invasion of the northern people in the Yellow River Basin, which was a group from the third period of Wangwan culture (Jin, 2010). Therefore, Chu’s agriculture was marked by the dual traits of northern and southern Chinese agriculture, comprising various crops and sophisticated planting patterns.

According to the archaeobotanical research carried out over the past two decades, the middle Yangtze River was one of the most important areas for paddy agriculture (Fuller, 2011; Zhao, 2014, 2018, 2019). However, little is known concerning the subsistence and dietary conditions of the commoners of Chu due to the space of the archaeobotanical and relevant specialist work. Much of the current knowledge is derived from later textual records such as *Chu Ci*, which is biased toward the lifeways of the aristocracy (Chang, 1979).

By analyzing the flotation results from the Wanfunao site (Figure 1), which is an early Chu culture site, the plant resources of the Chu people, especially crop resources, can be studied. The landform around the Wanfunao site was distinctly different from the adjacent Jianghan Plain in the east. The site is situated on a narrow alluvial plain of the Yangtze surrounded by low mountains while Jianghan Plain is much more open, flat, and with numerous lakes. In other words, the Wangfunao site was hillier than the east plain. Undoubtedly, it is worthwhile to investigate whether northern agricultural production was practiced in Chu, where rice cultivation has long been developed since the Neolithic (Zhao, 2014).

Previous archaeobotanical research in Hubei has largely focused on the eastern Jianghan Plain (Figure 1; Wu et al., 2010; Ng, 2011; Wang et al., 2011; Deng and Gao, 2012; Deng et al., 2013; Tang et al., 2014, 2016, 2017, 2019, 2020, 2021, 2022; Qin et al., 2017; Tian et al., 2019; Yang et al., 2020). The flotation results of the sites from the eastern region, such as Yejiamiao, Chengzishan, Qujialing, and Xiezidi, revealed that besides rice, which was a kind of stable crop, foxtail millet had been present in this area since the late Neolithic period, and by the Western Zhou Dynasty, it had gained significant importance (Wu et al., 2010; Deng et al., 2013; Tang, 2014; Tang et al., 2014, 2017; Yao et al., 2019). It is worth noting that at the Yejiamiao site, foxtail millet and rice spikelet base appeared in the same contexts or archaeological features (Wu et al., 2010).

Therefore, this study addresses a new case of archaeobotanical research in the western margin of the Jianghan Plain and compensates for the lack of archaeobotanical research in the historical period of China.

## The background of the Wanfunao site

### The natural environment of the site

**FIGURE 2:**
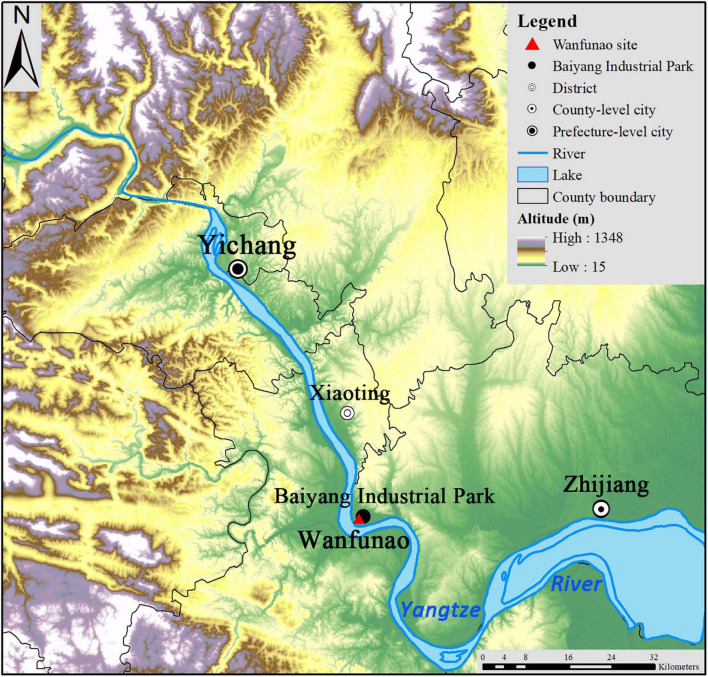
Regional landforms around Wanfunao site.

**FIGURE 3:**
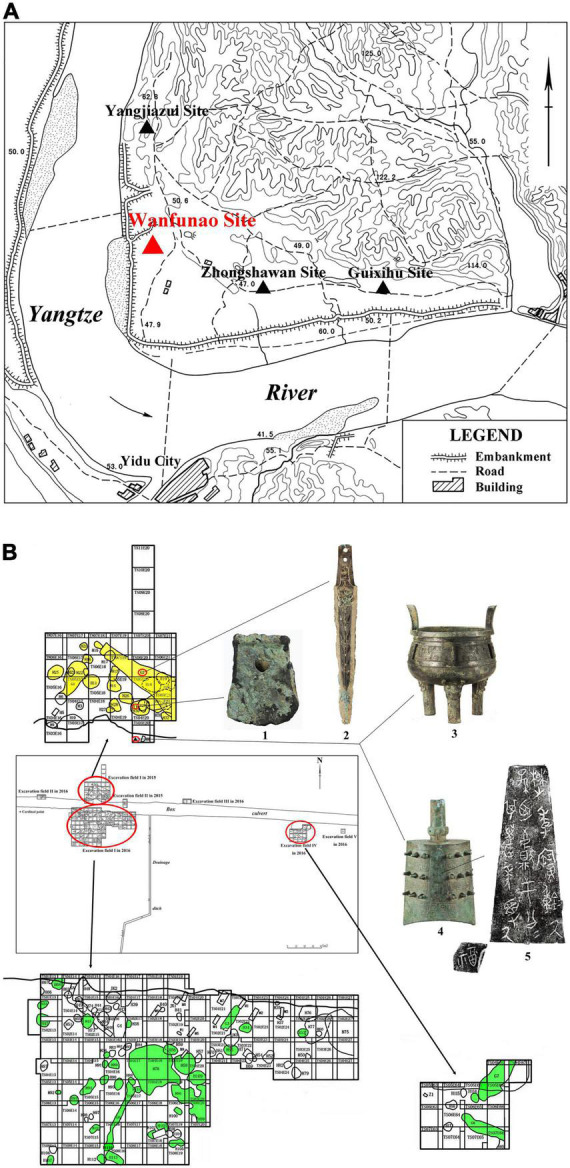
**(A)** The location of Wanfunao site and other surrounding sites in Baiyang Industrial Park. **(B)** Distribution of ruins and bronzes unearthed from Wanfunao. (1) bronze axe (Y1①:1), (2) bronze sword (G2②:1), (3) bronze tripod (TN03E20:13), (4) bronze bell (TN03E20:1), and (5) inscription of bronze bell (TN03E20:1).

The Wanfunao site (30°25′24.5′′N, 111°26′50.9′′E) is located in Baiyang Industrial Park, Yichang City, in the Hubei Province (Figures 2, 3A). The excavation area is close to the Yangtze River, with an area of about 56 ha, 67 km away from the Chu capital of Ying (郢) (Ji’nancheng 纪南城) at Jingzhou City (Figure 1 and Supplementary Figure 1; Wang et al., 2016). The Yangtze River in the Yichang section flows north to south, creating a narrow alluvial plain. There is a transition zone between the western Hubei mountainous region and the Jianghan plain. From west to east, there is a great disparity in terrain and altitude (35–2427 m.a.s.l.), as the region comprises mountains, hills, and plains (Hubei Provincial-Local Annals Compilation Committee, 1997). Summers are extremely moist due to the humid subtropical monsoon climate (Hubei Provincial-Local Annals Compilation Committee, 1997). Nowadays, cattle and sheep can be raised in the grasslands above 800 m.a.s.l., wheat and maize can be grown in the highlands, and rice is usually grown in the lowlands or plains (Hubei Provincial-Local Annals Compilation Committee, 1997).

### The excavation of the site

The Wanfunao site drew considerable scholarly attention in 2012 when 12 bronze bells and one bronze tripod were discovered during municipal construction (Supplementary Figure 2). The bells are believed to have belonged to Chu’s nobles owing to the inscription on the framed panel (Zheng/钲) carving “Chu Ji’s precious bell, the grandson cast the bronze bell to bless his grandfather” (楚季宝钟厥孙乃献于公公其万年受厥福) (Figure 3B; Huang and Zhao, 2016; Da, 2019). Based on typological analysis and textual research, ancient literal scholars Xueqin Li et al. believed that the bells belonged to the early period of the middle Western Zhou period to the late Western Zhou period (Huang and Zhao, 2016).

In 2013, a team of archaeologists from the Hubei Provincial Institute of Cultural Relics and Archaeology, the Department of Archaeology of Wuhan University, and the Yichang Museum has conducted preliminary field investigations at the Wanfunao site and its surrounding areas. Their investigations have confirmed the Wanfunao site and several ambient Zhou Dynasty sites such as Guixihu, Zhongshawan, and Yangjiazui (Figure 3A; Wang et al., 2015).

From 2015 to 2017, this team excavated the site scientifically and systematically (Supplementary Figures 3, 4). They arranged the trenches around the place where the bronzes were first found (Figure 3B). The excavation area was 0.25 ha, with ruins including ash pits, ash ditches, and pottery kilns. Further, they unearthed relics, such as stoneware, pottery, faience beads, a bronze sword with “double-dragon-heads,” and a bronze axe (Figure 3B and Supplementary Figure 5; Wang et al., 2016). Based on their work, they considered the site to be a large early Chu culture settlement (Wang et al., 2016).

**FIGURE 4:**
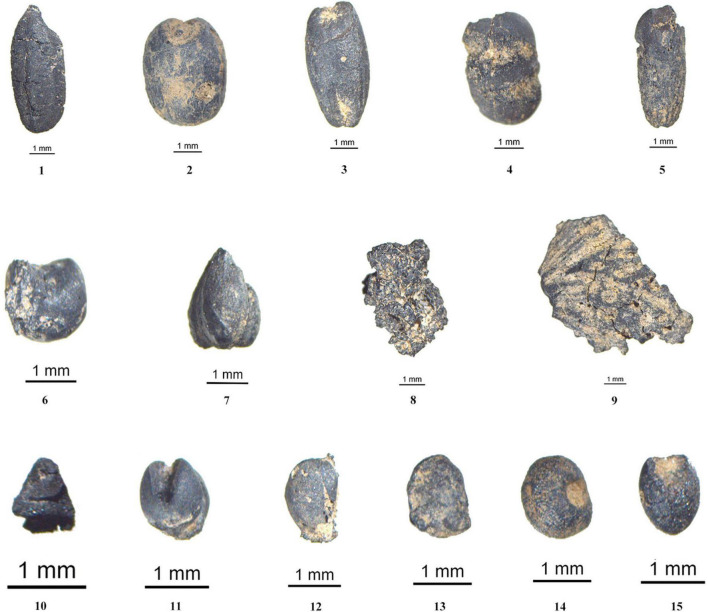
Images of Wanfunao plant remains unearthed in part. (1) *Oryza sativa*, (2) *Triticum aestivum*, (3) *Hordeum vulgare*, (4) *Vigna angularis*, (5) *Avena sativa*, (6) *Panicum miliaceum*, (7) *Fagopyrum esculentum*, (8) foxtail millet lump, (9) *Armeniaca mume*, (10) rice spikelet base, (11) *Setaria italica*, (12) *Melilotus* sp., (13) *Lespedeza* sp., (14) *Galium* sp., and (15) *Panicum bisulcatum*.

Ma et al. (2019) determined that the Wanfunao site likely existed from the late Zhou Dynasty to the middle Spring and Autumn period (983–775 cal. BC), based on the 14C dating results (Supplementary Table 1). This date spans approximately two hundred years.

## Materials and methods

To acquire accurate information on the distribution of plant remains as well as the people’s utilization of plants, soil samples were collected from ruins and strata from 2015 to 2017 (Zhao, 2010). In particular, 6,438 liters of soil out of 629 samples were collected. Finally, 89 merged soil samples are presented in Table 1 by placing the same samples into a group.

**TABLE 1:** Merged samples at the Wanfunao site from 2015 to 2017.

Ruins	Ash pit	Ash ditch	Pottery kiln	Layer	Total
Total	73	6	3	7	89

Flotation work was carried out by using two measuring buckets (10 L) fitted with two sifters (0.2- and 3.35-mm mesh) to collect carbonized plant remains and separate heavy fractions from light fractions (Zhao, 2010; Pearsall, 2015). Next, the floats were wrapped in a cotton cloth bag, labeled, and left to dry in the shade.

This study focused on the ancient plant seeds and fruit remnants from the light fractions. The Archaeobotanical Laboratory of the School of Cultural Heritage of Northwest University (Xi’an) was responsible for the identification and analysis of all samples. The stereo microscope (Nikon SMZ25) was used for observation, photography, and measurement.

## Results

**FIGURE 5:**
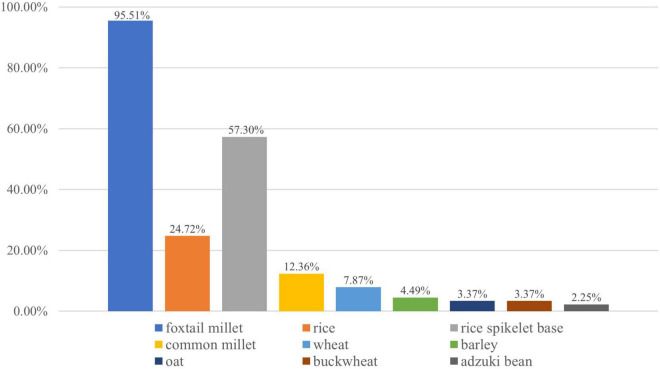
Ubiquity of crop remains at Wanfunao.

In all, 34,494 carbonized plant remains were found, including crop remains and non-crop remains. Among the crop remains and non-crop remains, there were 34,270 crop grains comprising 99.35% (Table 2, Figure 4, and Supplementary Table 2). Further, among crop remains, foxtail millet (*Setaria italica*) was the most abundant crop with the highest ubiquity recording at 95.51%. This was followed by rice (*Oryza sativa*), accounting for 24.72% ubiquity (Figure 5). Only 58 grains of other kinds of crops were found, such as common millet (*Panicum miliaceum*), bread wheat (*Triticum aestivum*), barley (*Hordeum vulgare*), oat (*Avena sativa*), buckwheat (*Fagopyrum esculentum*), and adzuki bean (*Vigna angularis*). These crops were diverse in species but low in quantity. Additionally, 5,141 rice spikelet bases accounting for 57.30% ubiquity, 763 foxtail millet lumps and one bread wheat rachilla were found (Figure 5). Table 2 provides the measurements of the intact crop grains.

**TABLE 2:** The quantity and measurement for crops at Wanfunao.

Family	Taxa	Total	Measurement (mm)	
			Average length	Average width
Poaceae	*Setaria italica*	28,105	1.177	1.11
	Foxtail millet lump	763		
	*Oryza sativa/*fragments	203	4.712	2.494
	Rice spikelet base	5,141		
	*Panicum miliaceum*	21	1.738	1.72
	*Triticum aestivum*/fragments	13	4.25	2.34
	Bread wheat rachilla	1		
	*Hordeum vulgare/*fragments	14		
	*Avena sativa/*fragments	4	4.23	1.68
Polygonaceae	*Fagopyrum esculentum*	3	1.84 (fragment)	2.38
Fabaceae	*Vigna angularis*	2		

A total of 224 wild seeds and seed fragments were identified from Wanfunao. These can be grouped into 24 categories, representing 18 plant families, such as Poaceae, Fabaceae, Lamiaceae, Polygonaceae, Cyperaceae, Chenopodiaceae, Potamogetonaceae, Trapaceae, Adoxaceae, Verbenaceae, Rubiaceae, Scrophulariaceae, Valerianaceae, Alangiaceae, Anacardiaceae, Rutaceae, Vitaceae, and Rosaceae (Figure 4, Table 3, and Supplementary Tables 3, 4). The taxonomic level of identification varied depending on the quality of the specimen, the amount of morphological distinguishability among species in a genus, and the availability of comparative specimens for all species of that genus/family. The wild plant remains accounted for only 0.65% of carbonized plant remains. In this study, only Fabaceae, which comprised most of the remains, was considered for analysis.

**TABLE 3:** The quantity and measurement for non-crops at Wanfunao.

Family	Genus/Species	Total	Measurement (mm)		
			Average length	Average width	Average diameter
Poaceae	*Digitaria sanguinalis*	10	1.12	0.58	
	*Setaria viridis*	6	1.22	0.73	
	*Panicum bisulcatum*	3	1.24	0.94	
	*Ischaemum barbatum*	5	1.33	0.66	
Fabaceae	*Melilotus* sp.	21	1.36	0.86	
	*Lespedeza* sp.	18	1.43	0.98	
	*Vicia sepium*	8			2.20
Lamiaceae	*Perilla frutescens*	24			1.20
Polygonaceae	*Rumex acetosa*	6	1.26	0.88	
	*Polygonum amphibium*	1			1.31
Cyperaceae	*Schoenoplectus juncoides*	1	1.38	1.39	
Chenopodiaceae	*Chenopodium album*	1			0.89
Potamo- getonaceae	*Potamogeton distinctus*	1	1.37	1.11	
Trapaceae	*Trapa* sp.	8			
Adoxaceae	*Sambucus javanica*	4	1.49	0.90	
Verbenaceae	*Vitex negundo* var. *heterophylla*	31	2.26	1.66	
Rubiaceae	*Galium* sp.	63			1.21
Scrophulariaceae	*Veronica polita*	1			1.05
Valerianaceae	*Patrinia scabiosifolia*	1	1.36	0.72	
Alangiaceae	*Alangium chinense* fragment	1	5.18	3.9	
Anacardiaceae	*Rhus chinensis*	1	2.18	2.62	
Rutaceae	*Zanthoxylum bungeanum*	1			2.20
Vitaceae	*Vitis* sp.	2	2.98	2.60	
Rosaceae	*Armeniaca mume* fragments	6			

## Discussion

### Agricultural economic characteristics of the early Chu people at Wanfunao

As shown in Figure 5, the crop assemblage was composed of indigenous Chinese crops, such as foxtail millet, broomcorn millet, rice, and buckwheat, and extraneous crops from Southwestern Asia, such as wheat and barley. Except for rice, all other crops were dryland crops. These crops were commonly grown in northern China. Both foxtail millet and rice were the critical grain from crop remains; foxtail millet was more ubiquitous than rice. Other crops, such as wheat, barley, oat, buckwheat, and adzuki bean, had less than 10% ubiquity.

**FIGURE 6:**
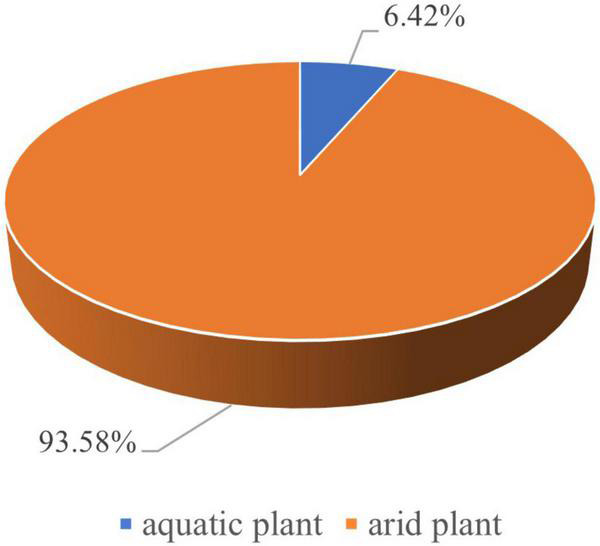
Pie chart recording the proportion of wild arid plant seeds and wild aquatic plant seeds at Wanfunao.

However, it must be noted that rice spikelet bases have been found together with a large amount of foxtail millet grains, suggesting the possibility that they were garbage discarded by people. Dryland wild seeds, which accounted for 93.58% from the flotation, also indicate a drought floristic habitat around the site (Figure 6). Therefore, the northern dryland crops, especially foxtail millet, must have been cultivated at the site. In other words, the Wangfunao people practiced mixed farming consisting of both rice planting and dryland crop planting.

### Adaptation to the compatible environment for multi-cropping at Wanfunao

The introduction of northern species at Wanfunao came with several seasonality challenges, for example, low water requirement versus high water requirement, long-day versus short-day. In this regard, three aspects, environmental advantages, plant adaptation, and human wisdom, influenced multi-cropping at Wanfunao.

Matched conditions for rice cultivation can be found in the Middle Yangtze River, where rice originated. The climate of Yichang City is influenced by the East Asian monsoon, which results in a hot season synchronized with the rainy phase, with over 800 mm of rainfall (Yichang Local Annals Compilation Committee, 2012; Supplementary Figure 6). The boundary between rice and dryland crops is 800 mm isohyet. Moreover, paddy fields in Yichang are distributed on the alluvial plains with a strong water holding capacity.

The watery environment was unsuitable for dryland crops, although the heat, temperature, and soil favored their growth (Encyclopedia of Chinese Agriculture Compilation Committee, 1991; Guan, 2011). Therefore, how and where the dryland crops grew at Wanfunao are the critical questions.

Nowadays, dryland crops in Yichang, such as wheat, maize, sweet potato (*Ipomoea batatas*), and potato, are more widely cultivated in the highlands. In this region, the transition zone between the upper and middle steps of the Chinese topography is a compatible environment for crop cultivation. The landform is characterized by plains, hills, and mountains (Figure 2). The alluvial plain along the Yangtze River is small, accounting for only 10.67%, whereas the arable area accounts for 137,300 ha, including 45.09% dryland. Meanwhile, the western and eastern regions by the side of the alluvial plain are mainly hills and mountains and account for 89.33%, whereas the arable area of hills and mountains accounts for 143,800 ha, including 83% dryland (Yichang Local Annals Compilation Committee, 2012). Such vast gradient landforms have several advantages such as well-draining capability and a climate that is cool enough to support moist-intolerant dryland crops, including millets and buckwheat (Zhou et al., 2018; Tang et al., 2019).

However, the millet planting area is limited in China due to the transformation of the structure of agricultural production, including in Yichang, over the past decades, but common wheat has always been an important crop. In the Yangtze River Basin, people may have begun wheat cultivation in paddy fields around Tang and Song dynasties (Guo, 1994). Before 1950, dryland wheat was more common because of the limitation of winter planting in Hubei Province and because paddy-field wheat cultivation was unacceptable to the residents (Hubei Provincial-Local Annals Compilation Committee, 1997). In recent years, the wheat planting area in paddy fields has seen an increase due to advances in agricultural technology. Until 1984, this area was almost equal to that of hills and mountains, covering 45,600 ha (Yichang Local Annals Compilation Committee, 2012). Thus, in the Zhou Dynasty, given the low level of agricultural technology and the limited arable area in the plains, dryland crop planting may have been carried out in the hills and mountains where the reclaimed drylands were popular.

Moreover, Yichang has a shorter daytime than the northern area during the spring equinox and autumn equinox. Wheat, barley, and oat are long-day crops, while others are short-day crops (Encyclopedia of Chinese Agriculture Compilation Committee, 1991; Guan, 2011). Based on the local phenological data in Yichang, winter wheat cultivation is overwhelming among wheat species (Table 4). Chinese historians consider the earliest wheat remains from the archaeological sites in China as winter wheat, hexaploidy, from Southwestern Asia (Han, 2013; Du, 2020), where the wheat is sensitive to photoperiod and vernalization (Ferrara et al., 1998). Following a long period of adaptation, mostly modern southern Triticeae crops have become winter species to that are insensitive to photoperiod and vernalization as the latitude decreases. Generally, following vernalization is a warmer climate with scarce freeze and frost in spring; southern wheat is insensitive to photoperiod and can succeed in earing up and flowering under the short-day situation (Jin, 1961). By Zhou Dynasty, wheat had been introduced to China for over a thousand years, and humans had sufficient knowledge of winter wheat cultivation. Owing to the stronger adaptability of hexaploidy wheat, novel wheat cultivars might have better matched the photoperiodic differences between the north and the south in Zhou Dynasty. Archaeologists anticipate the genetic analysis of wheat remains, although such an analysis is sufficiently difficult in the normal sedimentary context.

**TABLE 4:** Contemporary phenological phase of staple crops in Yidu county-level city, Yichang city, Hubei Province.

Phase	Sowing time	Flowering time	Harvest time	Growing season
Crop type				
Early rice	20/3–12/4	27/6–8/7	20/7–21/7	110–120
Mid-season rice	12/4–22/4	8/8–20/8	10/9–20/9	130–150
Late rice	3/6–24/6	5/9–20/9	10/10–25/10	130–145
Wheat	15/10–15/11	8/4–20/4	10/5–24/5	200
Barley	1/11–30/11	28/6–15/7	5/5–15/5	180
Buckwheat	25/8–10/9	20/9–10/10	25/10–5/11	85

The situation in ancient times should have been similar to what it is today. Multi-crop assemblage unearthed in Wanfunao suggests that the ancestors had a profound knowledge of the requirements of different types of crops, including knowledge related to the landform, temperature, light, water, and soil. It was reasonable for the early Chu people residing in the Wanfunao site to utilize paddy fields for rice planting and reclaim gradient hills and mountains for various dryland crops. The pattern, referred to as the mountains-hills-plains subsistence system, should be a mode that have been integrated with the terrain differences between eastern and western China, and bridged the climatic divergence between southern and northern China. In this compatible environment, the Chu people reclaimed different types of lands to ensure sufficient agricultural production. This situation is similar to that of the hilly area of the northwestern margin of the Jianghan Plain, especially the cases from the sites in Gouwan, Qinglongquan, Dasi, Mulintou, and Jijiawan. The flotation results of these sites have revealed that prehistoric southerners who settled in the hilly or mountainous environment may have performed multi-crop farming, relying on both rice cultivation and millet cultivation, especially within the high ubiquity of millets (Ng, 2011; Wang et al., 2011; Tang et al., 2016, 2019; Tian et al., 2019). Thus, with the southward spread of dryland crops as well as the adaptation of these crops to the southern environment, multi-cropping gradually increased at Wanfunao in southern China.

Besides crop remains, a few plum stones (*Armeniaca mume*) and grape seeds (*Vitis* sp.) have also been found, indicating that the Wanfunao ancestors grew fruits in the hilly regions. Similar activities were performed in other early Chu culture sites located in the valley surrounded by hills and mountains (Figure 1). In other words, their subsistence was dependent on animal and plant resources available in the compatible environment (Da and Li, 2012).

### Multi–cropping prevalence in southern China

Dryland crops such as foxtail millet had been utilized since the late Neolithic period (∼5000 cal. BP) in South China, e.g., the Qujialing site, where rice farming was common. AMS radiocarbon dating indicates that the foxtail millet at Qujialing is China’s earliest known, securely dated foxtail millet [5584–5449 cal. BP (75.3%)] (Yao et al., 2019). Ancient millets have been found in the prehistoric sites of southern China, i.e., Zhongba site, Chongqing (4500–3750 cal. BP) (Zhao and Flad, 2013), Nanshan site, Fujian (5300–4400 cal. a BP) (Yang et al., 2018), Pingfengshan and Huangguashan sites, Fujian (4300–3500 cal. BP) (Deng et al., 2018), Hulushan site, Fujian (4000–3500 cal. BP) (Fu et al., 2016), and Nanguanlidong site, Taiwan (4800–4200 cal. BP) (Tsang et al., 2017). This suggests that ancient southern Chinese residents performed efficient and practical activities to plant dryland crops by taking advantage of the mountains and hills. To understand the development of crop varieties and the maturity of the multi-crop agriculture system from the late Neolithic period to the Zhou Dynasty, spanning approximately 2,000 years, further information is required. This study revealed that the multi-crop farming system had been carried out in southern Chinese settlements, including Wanfunao, by early Western Zhou.

**FIGURE 7:**
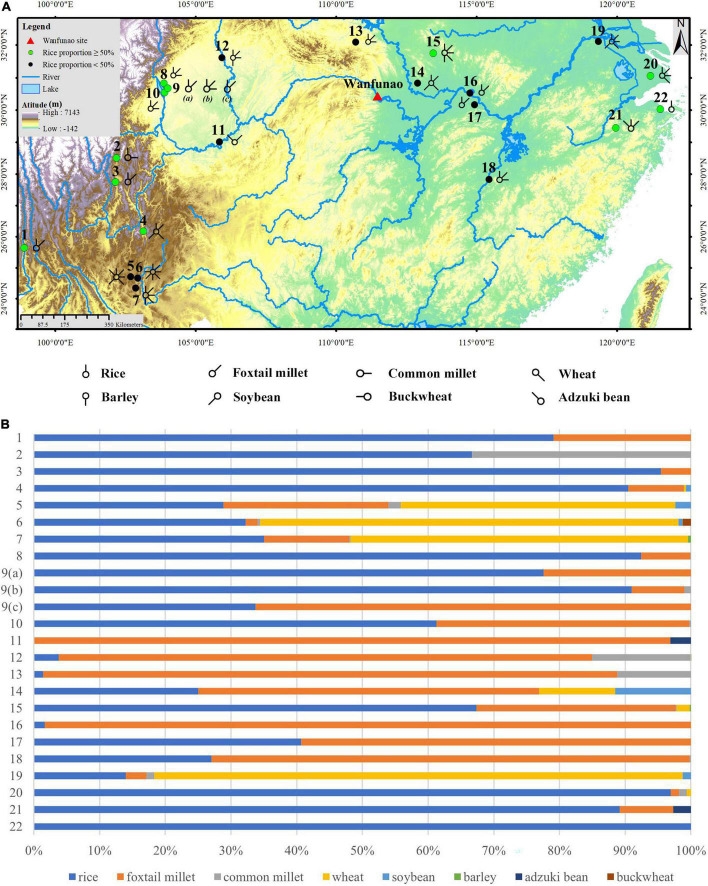
Crop assemblage and proportion of Zhou Dynasty in the south of Yangtze River. **(A)** Crop assemblage; **(B)** carbonized crop proportion. (1) Shilinggang, (2) Gaopo, (3) Shapingzhan, (4) Yubeidi, (5) Heposuo, (6) Xueshan, (7) Guangfentou, (8) Boluocun, (9) Jinsha (a) Jinniu district No. 5, (b) Sacrificial area, and (c) Yangguangerqididai, (10) Sanguantang, (11) Handongcheng, (12) Zhengjiaba, (13) Jijiawan, (14) Qujialing, (15) Miaotaizi, (16) Chengzishan, (17) Xiezidi, (18) Niucheng, (19) Dingjiacun, (20) Guangfulin, (21) Shangshan, and (22) Yushan.

According to available statistics, paleoethnobotanical research has been conducted in approximately 22 Zhou Dynasty sites in southern China. Besides rice, other crops, such as foxtail millet, broomcorn millet, wheat, barley, soybean, and buckwheat, were all found by floatation (Figures 7A,B). Figure 7A depicts the dominance of rice/millet in the crop assemblage. It is evident from Figure 7B that the sites with rice proportions exceeding 50% were primarily found in the lower reaches of the Yangtze River and the Ancient Dian Kingdom in Yunnan Province. Consequently, the multi-crop farming system existed in the southern Yangtze River valley as well as in northern China, where Zhou’s capital was located. A mosaic of mountains, hills, and plains characterized the topography of southern China, which supported the various ways in which ancient people engaged in agriculture. Ancient inhabitants of the Zhou Dynasty were accustomed to different crop cultivation and adapted their production to the local circumstance depending on their compatible environment.

The multi-crop farming system in southern China, especially in the Yangtze River valley, may have been related to social complexity considering the many advantages of the system. These advantages included raising the total value of agricultural output, reducing natural disasters, and increasing the diversity of crops (Zhao, 2005, 2011). It was under the influence of such a cropping system that the ancient southern societies of China further developed. Early Chu farmers, who practiced multi-cropping, cultivated a wide selection of crops, such as foxtail millet, broomcorn millet, rice, wheat, barley, oat, buckwheat, and adzuki bean. Their cultivation habits depict the diversity and development of early Chu agriculture.

### The ways of plant utilization

According to the analysis conducted in this study, millets, rice, and other crops unearthed at the Wanfunao site were predominantly from ash pits where ancient people discarded their garbage. The crops found in ash pits provide important insight into the ways of plant utilization (Zhong et al., 2020).

Plant ubiquity reflects ancient people’s preferences related to plant utilization (Zhao, 2010). This study found that foxtail millet at Wanfunao had a ubiquity of over 90%, the ubiquity of rice spikelet base was 57.30%, whereas rice had a ubiquity of only 24.72%. These findings indicate that the Wanfunao people had sufficient access to agricultural products. The presence of “foxtail millet grain/rice spikelet base” in the site may be related to forage, which was composed of foxtail millet grains and rice straw.

Foxtail millet was a kind of shared crop utilized by both humans and animals. It has high yields, high quality, and a sweet taste. The grass, in its dry, fresh, or ensiled form, is nutrient-rich with good palatability; therefore, it was a suitable alternative forage source for livestock and poultry in northern China (Shanxi Academy of Agricultural Sciences, 1987). The straw and grains of foxtail millet were usually mixed as forage for horses, cattle, and sheep, whereas pigs, chickens, and other animals were fed grains and chaff (He, 2010). Rice spikelet bases attached to rice husks were a by-product of rice processing (Wu et al., 2010). Consequently, the assemblage of “foxtail millet grain/rice spikelet base” may have been used for animal husbandry.

Furthermore, the early Chu people at Wangfunao are believed to have fed certain legumes to livestock, including *Melilotus* sp. and *Lespedeza* sp., both of which were fine fodders with fresh stems and tender leaves and were rich in nutrition (Li et al., 2004). It should be noted that both *Melilotus* spp. and *Lespedeza* spp. were found at 39 sites in the Shaanxi, Henan, and Shandong provinces of northern China. This means that these two legumes were utilized for raising livestock even before Qian Zhang brought alfalfa (*Medicago sativa*) to China during the Han Dynasty. However, since zooarchaeological research at the Wanfunao site is still a work in progress, further research is required to support this view, especially research involving carbon and nitrogen isotope analyses.

## Conclusion

The cereal-rich diet of the upper-class people belonging to the Chu region is well portrayed in *Chu ci*, “Your household pays homage at your shrine. With all kinds of food and wine, eh! With yellow millet, early wheat and rice. Offered in your sacrifice, eh!” (室家遂宗, 食多方些. 稻粢穱麦, 挐黄粱些) (Xu, 2012). The Wanfunao site was a large Chu settlement in the Zhou Dynasty. At this site, bronze bells were unearthed, representing the superior status of the people. The presence of crop remains at this site, such as foxtail millet, broomcorn millet, rice, wheat, barley, oat, buckwheat, and adzuki bean, confirm, to some extent, the description of the nobiliary diet in *Chu ci*.

The dispersal of crops resulted from regional communication. As the society developed, rice from southern China, millets from northern China, and wheat and barley from Southwestern Asia were exchanged through different cultural activities, such as war and trade. The Wanfunao people consumed both rice and dryland crops. Their diet structure influenced by the dry farming performed in the northern regions closely resembled the varied arable lands around the Wanfunao site. Plains and adjacent hills and mountains were well-known among the people as ideal locations for growing a variety of crops. The compatible environment of these landforms facilitated the cultivation of dryland crops in southern China, such as millets and wheat, which successfully adapted to the new environment and played a key role within the agricultural structure of the southern region. The multi-crop farming system had been practiced in the southern region of China since the late Neolithic Age and became popular by the end of the Zhou Dynasty. This may have been because the southern people inhabitants became increasingly acquainted with their habitat and learned to exploit different agricultural landforms for their agriculture. Thus, the diverse agricultural practices of the early Chu people were essential to the further development and prosperity of the Chu state. Additionally, the various plant resources, including foxtail millet, rice, *Melilotus* sp., and *Lespedeza* sp., may have been devoted to raising animals as well.

The Wanfunao site was an important Chu settlement among other contemporary sites in China. This paleoethnobotanical research on the site provides groundbreaking perspectives that can facilitate further in-depth research on the agriculture and diet of the people belonging to the Chu settlement. To fully reveal the livelihood pattern of the early Chu people, comprehensive studies, such as zooarchaeological research, must be conducted in the future, and flotation results must be acquired from other Chu sites as well.

## Data availability statement

The original contributions presented in this study are included in the article/Supplementary material, further inquiries can be directed to the corresponding author.

## Author contributions

RY and LT conceived of and designed the study, analyzed the data, and wrote the manuscript. LT, DZ, WH, and YL collected the data. WH and YL took charge of the excavation work. All authors have contributed to the manuscript and approved the submitted version.
