# Supplementary material for: Reclamation in southern China: The early Chu’s agriculture revealed by macro-plant remains from the Wanfunao site (ca. 1000–770 BCE)
Source: Front Plant Sci. 2022 Aug 2;13:942366. doi: 10.3389/fpls.2022.942366 (PMC9379102; doi:10.3389/fpls.2022.942366)
Supplement: Supplementary file 1 [file Data_Sheet_1.pdf]

frontiers

Supplementary Material

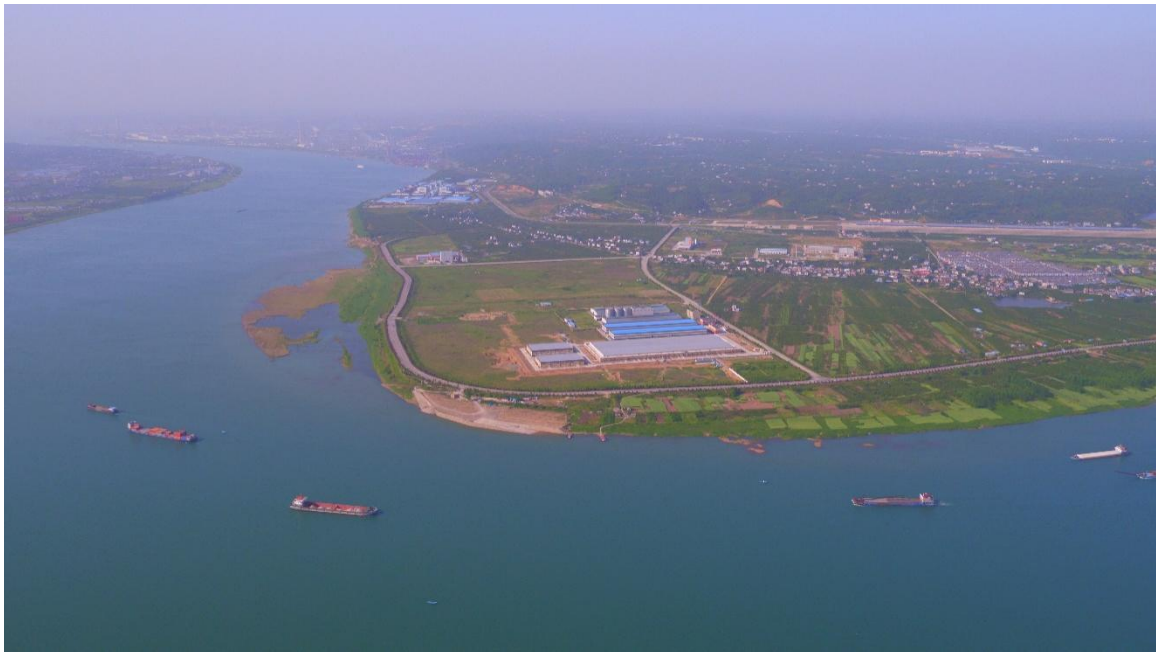

Supplementary Figure 1. Aerial image of Wanfunao (from southwest-northeast). Photo by Wenxin Huang.

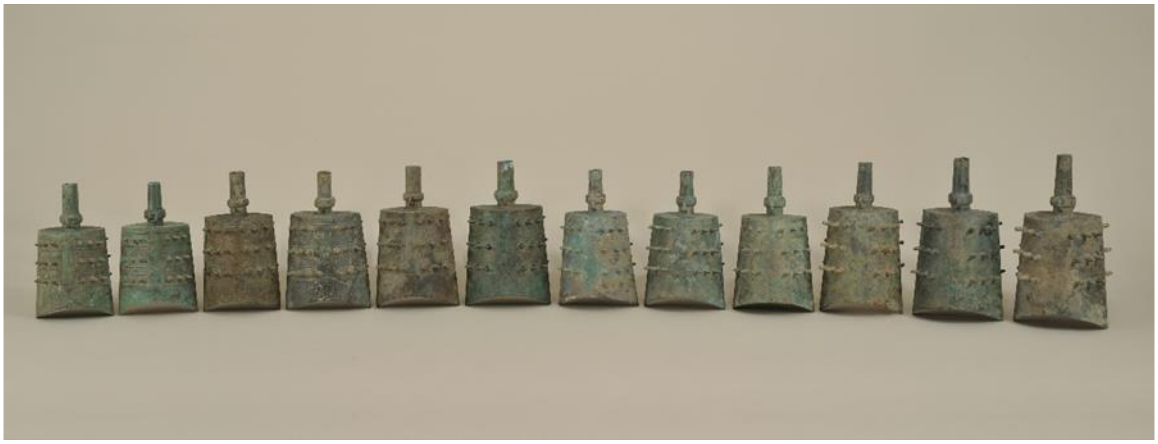

Supplementary Figure 2. Photograph of the bronze bells unearthed from Wanfunao (TN03E20: 1~12). Photo by Wenxin Huang.

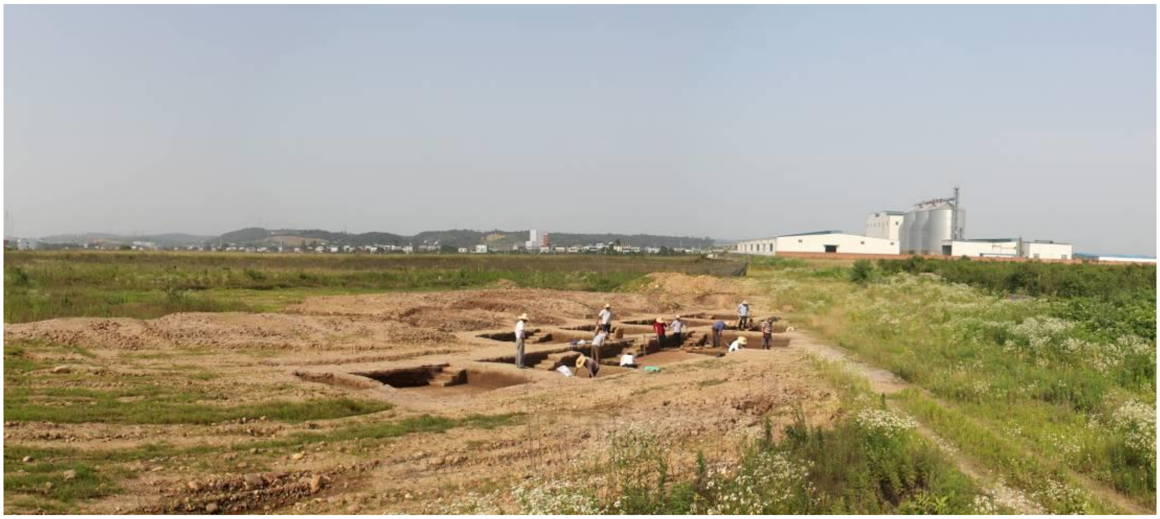

Supplementary Figure 3. Excavation of Wanfunao during 2015. Photo by Wenxin Huang.

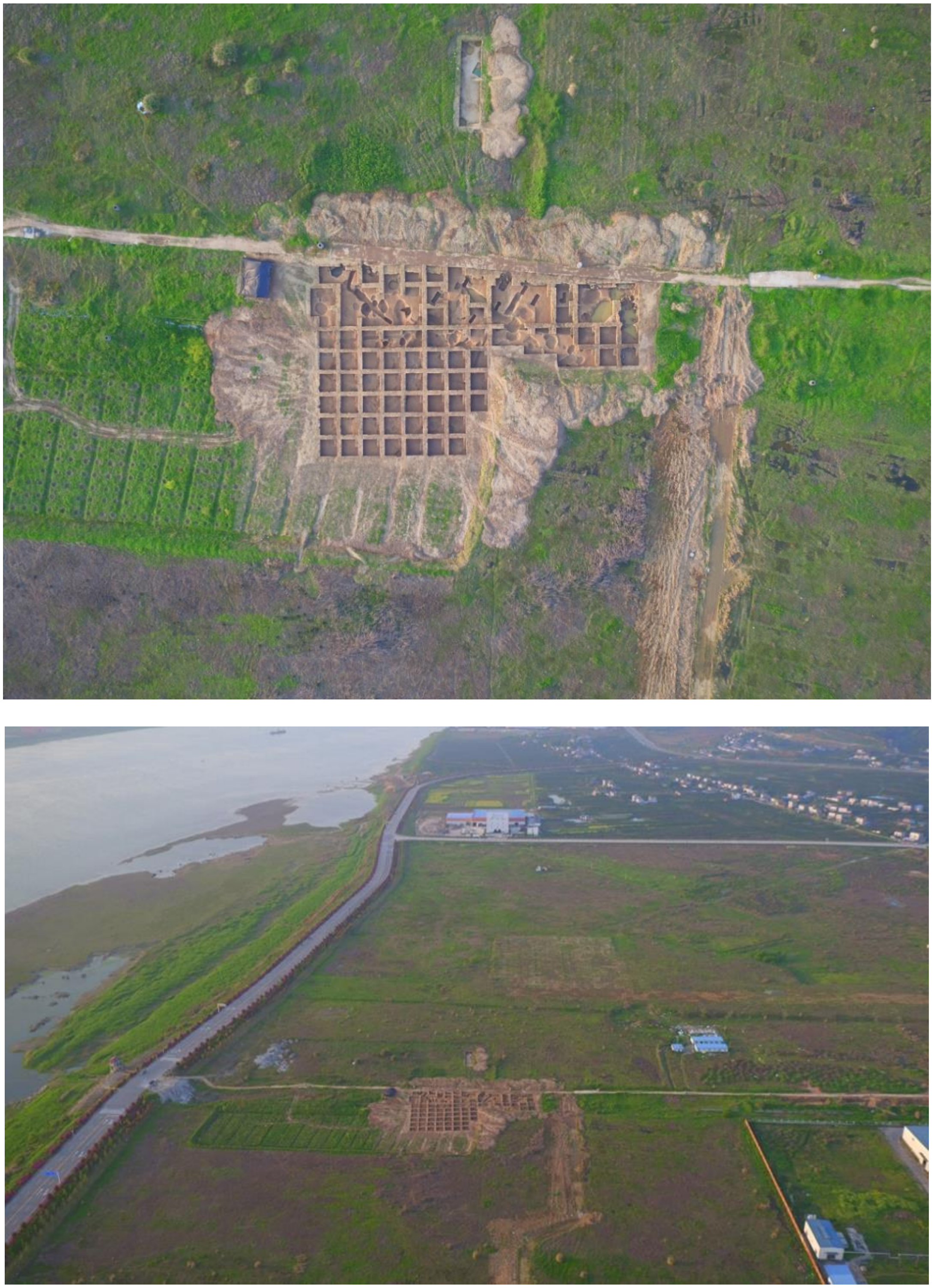

Supplementary Figure 4. Aerial images of the excavation field I in 2016 at Wanfunao. Photos by Wenxin Huang.

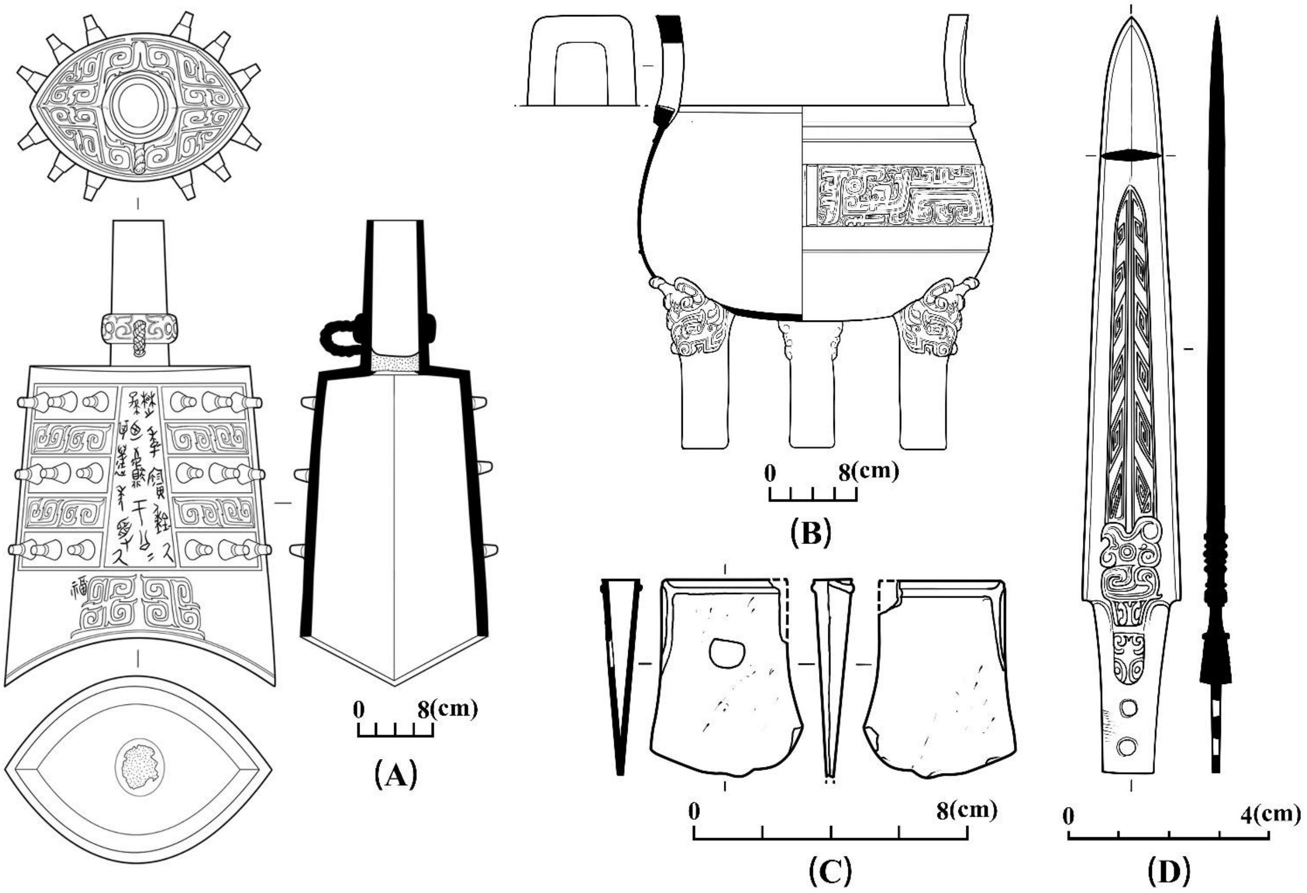

Supplementary Figure 5. Line drawing of the bronzes unearthed from Wanfunao. Figure by Chao Wang and Deming Fu. **(A)**bronze bell (TN03E20:1); **(B)**bronze tripod (TN03E20:13); **(C)**bronze axe (Y1①:1); **(D)**bronze sword (G2②:1)

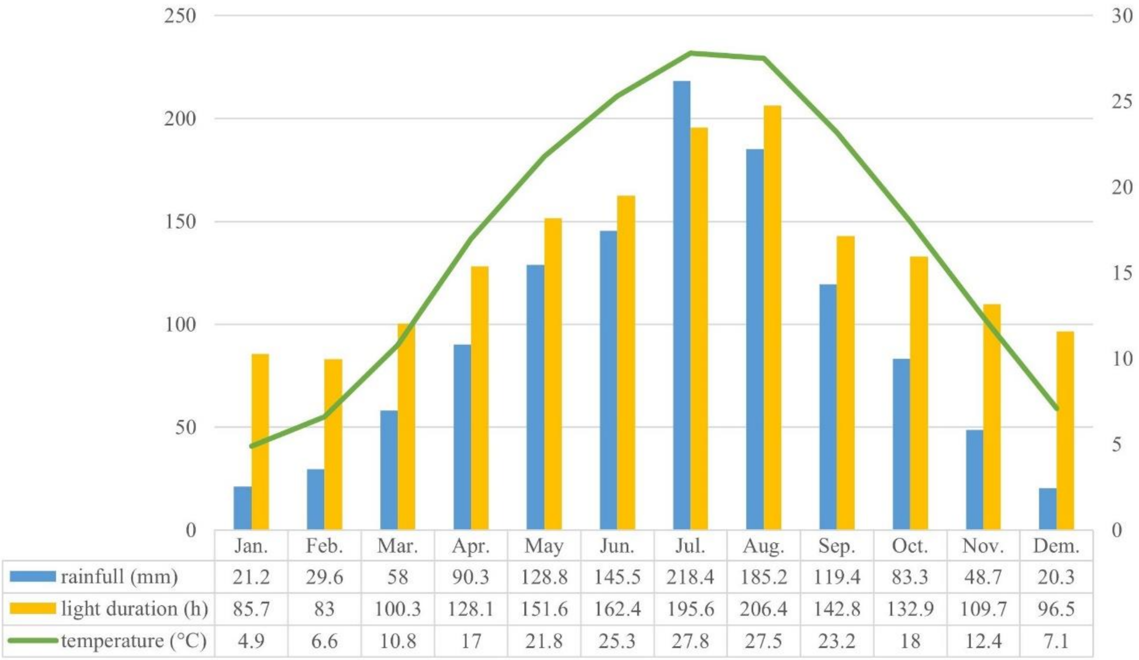

Supplementary Figure 6. Average rainfall, sunshine duration and temperature per year of Yichang City.

Supplementary Table 1. Radiocarbon dates for several carbonized rice and foxtail millet remains from Wanfunao Site. The data carried out in the previous research (9) and had been calibrated using the IntCal20 calibration curve.

Lab No.	Dated Material	Context	^{14}C age （BP）	Calibrated age (cal B. P.)	
				1δ-range (68.2%)	2δ-range (95.4%)
BA172410	Rice	H11①	2630±30	2761-2739(68.26%)	2777-2724(95.41%)
BA172415		H15	2765±25	2914-2909(2.27%)<br>2879-2842(35.84%)<br>2825-2785(30.03%)	2934-2778(95.4%)
BA172416		H16③	2680±40	2845-2820(21.54%)<br>2789-2750(46.61%)	2854-2742(95.42%)
BA172419		H25	2670±30	2838-2832(4.84%)<br>2782-2749(63.35%)	2847-2809(21.07%)<br>2800-2742(74.32%)
BA172420		H26①	2695±25	2843-2823(23.18%)<br>2786-2757(44.98%)	2847-2755(95.43%)
BA172421		H31①	2705±25	2845-2818(32.19%)<br>2790-2761(35.93%)	2849-2758(95.43%)
BA172424		G2②	2585±40	2757-2706(65.93%)<br>2627-2621(2.24%)	2768-2694(70.89%)<br>2638-2613(6.87%)<br>2593-2510(17.12%)<br>2507-2500(0.51%)
BA172417	Foxtail millet	H17②	2640±40	2776-2736(68.29%)	2847-2810(10.97%)<br>2799-2721(84.43%)
BA180802		G8③	2760±25	2876-2841(32.96%)<br>2826-2784(35.23%)	2931-2893(11.76%)<br>2890-2775(83.63%)
BA180805		H96	2765±25	2914-2909(2.27%)<br>2879-2842(35.84%)<br>2825-2785(30.03%)	2934-2778(95.4%)
BA180808		XH1②	2515±35	2723-2694(14.64%)<br>2638-2613(12.51%)<br>2592-2511(40.41%)<br>2506-2503(0.63%)	2739-2489(95.41%)
BA180809		G7②	2870±30	3059-3011(26.4%)<br>3009-2953(41.78%)	3135-3130(0.51%)<br>3104-3096(0.9%)<br>3075-2877(93.98%)
BA180811		G8②	2785±35	2948-2847(68.22%)	2963-2779(95.41%)
BA180814		H78③	2770±35	2925-2900(13.47%)<br>2884-2842(31.17%)<br>2825-2785(23.55%)	2953-2777(95.41%)
BA180819		G7②	2870±50	3070-2927(64.06%)<br>2898-2885(4.14%)	3155-2864(95.4%)

Supplementary Table 2. Total crop remains counts, contexts, and liters of soil at Wanfunao.

**No.**	**Contexts**	**Vol. liters**	**Domesticates**														
			Setaria italica	*Panicum miliaceum*	foxtail millet lump	*Oryza sativa*	*Oryza sativa* fragments	rice spikelet base	*Triticum aestivum*	*Triticum aestivum* fragments	bread wheat rachilla	*Hordeum vulgare*	*Hordeum vulgare* fragments	*Avena sativa*	*Avena sativa* fragments	*Fagopyrum esculentum*	*Vigna angularis*
1	G1①	39	670				1	13		1							
2	G1②	21	109			2	3	153									
3	G1③	20	2					1									
4	G2①	141.5	142	1			2	48		1							
5	G2②	86	277			1		90									
6	G2③	40	151					22									
7	Y1①	11	30					2									
8	Y1②	11	66					85									
9	Y1③	10.5	94					6									
10	H11①	1128	2014	2		1	14	399	1				1	1	1	1	
11	H13①	1248	7968	6		5	54	625	1	5	1		5			1	
12	H13②	18.5	96				1	22									
13	H14①	1485	5447	2			20	649	1	1		1	6		1		1
14	H14②	797	4452	4		5	22	932		1					1	1	
15	H15 southern part	155.5	430				6	20									
16	H15①	88	560				3	8									
17	H15②	39	133	1				6									1
18	H16①	20.5	34	1				8									
19	H16②	19	45					10									
20	H16③	14	288				7	696									
21	H17①	30	118					2									
22	H17②	24.5	132					25									
23	H17③	20	88					70									
24	H18① western part	5	5														

25	H18②	5	2					4									
26	H18③	8	25	1				9									
27	H20	4	29					7									
28	H21	15	68					8									
29	H22	14	6				1	1									
30	H23	14.5	15														
31	H24②	16	418			2	9	466									
32	H24③	7	20					98									
33	H25	20	384				14	286									
34	H26	9	156			1	2	30									
35	H27	8.5	12														
36	H28	8	16					19									
37	H29	15.5	22					84									
38	H30	7	44			1	2	99									
39	H31①	9	80				3	21									
40	H32	10	58					4									
41	G3①	6															
42	G3②	7	70		3												
43	G6	35	33		5												
44	G7②	156	1998		476		3	14					1				
45	G8②	116.5	222		34			8									
46	G8③	27	46					6									
47	H33②	9	16		10												
48	H34	10.5	10														
49	H36	6	34		4												
50	H38②	6	3														
51	H45②	9	6		2												
52	H47①	10	34			2	4	22									
53	H53	7	36	1				4									
54	H56②	6	6					10									
55	H58	5	16		3			2									

56	H65	8	4		6												
57	H68	17	46	1	5												
58	H78①	36.5	41	1	4			1									
59	H78②	16	8		1			3									
60	H78③	10	62		15												
61	H88	7	38														
62	H89	9	23		2			2									
63	H90	15.5	26		21												
64	H91	7	14		5				1								
65	H92	7			2												
66	H93	17	27		11												
67	H94	8	23					38									
68	H95	10.5	14		10												
69	H96	38	127		28		1										
70	H100	5	4														
71	H101	6	2														
72	H102	2															
73	H103	20	48		35												
74	H105	4.5	6		2												
75	H107	8.5	19		5			1									
76	H109③	7			2												
77	H110	12	28		1												
78	H111	8	25		4												
79	H112	18	40		10												
80	H113	7	20		5												
81	H114	11	57		12												
82	H123	11	15		6			1									
83	TS04E18③	6	21		14												
84	TS04E19④	4	3		2												
85	TS04E19③	5	1		1			1									

86	TS06E20③	5	41		11												
87	TS06E20④	15	30		6												
88	TS07E66③	11	18														
89	XH1②	7	38			3	8										
**Total**		**6438**	**28105**	**21**	**763**	**23**	**180**	**5141**	**4**	**9**	**1**	**1**	**13**	**1**	**3**	**3**	**2**

Supplementary Table 3. Total seed counts, contexts of non-crop remains at Wanfunao, including Poaceae, Fabaceae, Lamiaceae, Polygonaceae, Cyperaceae, Chenpodiaceae and Potamogetoaceae.

No.	Contexts	Poaceae				Fabaceae			Lamiaceae	Polygonaceae		Cyperaceae	Chenopodiaceae	Potamogetonaceae
		Digitaria sanguinalis	*Setaria viridis*	*Panicum bisulcatum*	*Ischaemum barbatum*	*Melilotus* sp.	*Lespedeza* sp.	*Vicia sepium*	*Perilla frutescens*	*Rumex acetosa*	*Polygonum amphibium*	*Schoenoplectus juncoides*	*Chenopodium album*	*Potamogeton distinctus*
1	G1①					1								
2	G1②			2										
3	G1③													
4	G2①	1				1	1		1					
5	G2②													
6	G2③													
7	Y1①													
8	Y1②								1					
9	Y1③													
10	H11①	2	1			3	4	1	2	1				
11	H13①		2	1		6	5			1		1		1
12	H13②													
13	H14①	4				5	8	5	2	3				
14	H14②	2	1			1			4					
15	H15 southern part					1			6					
16	H15①								8					
17	H15②													
18	H16①												1	
19	H16②													
20	H16③										1			
21	H17①													
22	H17②													
23	H17③													
24	H18① western part													

25	H18②													
26	H18③													
27	H20													
28	H21													
29	H22													
30	H23													
31	H24②													
32	H24③													
33	H25		1		5									
34	H26													
35	H27													
36	H28													
37	H29													
38	H30													
39	H31①													
40	H32													
41	G3①													
42	G3②													
43	G6													
44	G7②	1						1		1				
45	G8②													
46	G8③													
47	H33②													
48	H34													
49	H36													
50	H38②													
51	H45②													
52	H47①		1											
53	H53													
54	H56②													
55	H58													

56	H65													
57	H68													
58	H78①													
59	H78②													
60	H78③													
61	H88													
62	H89					3		1						
63	H90													
64	H91													
65	H92													
66	H93													
67	H94													
68	H95													
69	H96													
70	H100													
71	H101													
72	H102													
73	H103													
74	H105													
75	H107													
76	H109③													
77	H110													
78	H111													
79	H112													
80	H113													
81	H114													
82	H123													
83	TS04E18③													
84	TS04E19④													
85	TS04E19③													

86	TS06E20③													
87	TS06E20④													
88	TS07E66③													
89	XH1②													
**Total**		**10**	**6**	**3**	**5**	**21**	**18**	**8**	**24**	**6**	**1**	**1**	**1**	**1**

Supplementary Table 4. Total seed counts, contexts of non-crop remains at Wanfunao, including Trapaceae, Adoxaceae, Verbenaceae, Rubiaceae, Scrophulariaceae, Valerianaceae, Alangiaceae, Anacardiaceae, Rutaceae, Vitaceae and Rosaceae.

**No.**	**Contexts**	**Trapaceae**	**Adoxaceae**	**Verbenaceae**	**Rubiaceae**	**Scrophulariaceae**	**Valerianaceae**	**Alangiaceae**	**Anacardiaceae**	**Rutaceae**	**Vitaceae**	**Rosaceae**
		Trapa sp.	*Sambucus javanica*	*Vitex negundo* var. *heterophylla*	*Galium* sp.	*Veronica polita*	*Patrinia scabiosifolia*	*Alangium chinense* fragment	*Rhus chinensis*	*Zanthoxylum bungeanum*	*Vitis* sp.	*Armeniaca mume* fragments
1	G1①											
2	G1②			1								
3	G1③											
4	G2①			1	1							
5	G2②											
6	G2③											
7	Y1①			1								
8	Y1②			3								
9	Y1③			1								
10	H11①			1	9				1	1		
11	H13①	6	1	4	17		1					1
12	H13②											
13	H14①	2		1	29	1		1				1
14	H14②			13							1	
15	H15 southern part											
16	H15①		1									
17	H15②											
18	H16①											
19	H16②											
20	H16③											
21	H17①											
22	H17②		1									
23	H17③											
24	H18①											

	western part											
25	H18②											
26	H18③											
27	H20											
28	H21		1									
29	H22											
30	H23											
31	H24②											
32	H24③											
33	H25											
34	H26			2							1	4
35	H27			3								
36	H28											
37	H29											
38	H30											
39	H31①											
40	H32											
41	G3①				1							
42	G3②											
43	G6											
44	G7②				4							
45	G8②											
46	G8③											
47	H33②											
48	H34											
49	H36											
50	H38②											
51	H45②											
52	H47①											
53	H53											
54	H56②											

55	H58											
56	H65											
57	H68											
58	H78①											
59	H78②											
60	H78③											
61	H88											
62	H89											
63	H90											
64	H91											
65	H92											
66	H93											
67	H94											
68	H95				1							
69	H96											
70	H100											
71	H101											
72	H102											
73	H103				1							
74	H105											
75	H107											
76	H109③											
77	H110											
78	H111											
79	H112											
80	H113											
81	H114											
82	H123											
83	TS04E18③											
84	TS04E19④											

85	TS04E19③											
86	TS06E20③											
87	TS06E20④											
88	TS07E66③											
89	XH1②											
**Total**		**8**	**4**	**31**	**63**	**1**	**1**	**1**	**1**	**1**	**2**	**6**

Reference

Ma, R. J., Cui, J. F., Huang, W. X., Shi, D. Y., and Xiang, G. H. (2019). Scientific analysis of Copper Wares from Wanfunao Site in Yichang and related archaeological problems. *Jianghan Archaeology* 5, 121-130.

Yichang Local Annals Compilation Committee (2012). *Yichang Local Annals (1979-2000)*. Beijing: China Local Records Publishing, 143, 149.
